# Chlorate of Potash as a Tooth Powder

**Published:** 1896-04

**Authors:** 


### Chlorate of Potash as a Tooth Powder.

Unna recommends chlorate of potash as a tooth powder, and at the same time as an excellent disinfectant for the oral cavity. The brush is dipped into the powder as into any other powder, and the teeth are brushed lightly, the powder forming a paste with the saliva. Immediately afterward the mouth is rinsed out with water. For some time a salty taste remains which is not unpleasant. Unna knows of no remedy which abates bad breath due to decomposition of food between the teeth, and in the pharynx after remaining in the cavities over night. He had many such cases that sought his advice and all were cured by above treatment. There are cases, though, when a sore mouth or a fissure becomes very painful if the pure chlorate of potash is used, and in those cases he recommends a paste to be made of carbonate of lime, rhizoma, iridis, soap and glycerine equally mixed, then add the chlorate of potash to make a 50 percent paste.

